# Multistate Outbreak of SARS-CoV-2 B.1.1.529 (Omicron) Variant Infections Among Persons in a Social Network Attending a Convention — New York City, November 18–December 20, 2021

**DOI:** 10.15585/mmwr.mm7107a3

**Published:** 2022-02-18

**Authors:** Sarah E. Smith-Jeffcoat, Mary A. Pomeroy, Sadia Sleweon, Samira Sami, Jessica N. Ricaldi, Yonathan Gebru, Brianna Walker, Shane Brady, Matthew Christenberry, Stephen Bart, Johanna Vostok, Stephanie Meyer, Scott Seys, Amanda Markelz, Nicole Ditto, Valerie Newbern, Franda J. Thomas, Deepam Thomas, Enrico Cabredo, Stephanie Kellner, Vance R. Brown, Jacqueline E. Tate, Hannah L. Kirking

**Affiliations:** ^1^CDC COVID-19 Emergency Response Team; ^2^Arizona Department of Health Services; ^3^Pima County Health Department, Tucson, Arizona; ^4^Connecticut Department of Public Health; ^5^Epidemic Intelligence Service, CDC; ^6^Massachusetts Department of Public Health; ^7^Minnesota Department of Health; ^8^Missouri Department of Health and Senior Services; ^9^St. Louis Department of Health, St. Louis, Missouri; ^10^New Jersey Department of Health; ^11^Monmouth County Health Department, Freehold, New Jersey; ^12^Virginia Department of Health.

On December 2, 2021, the Minnesota Department of Health (MDH) notified CDC of a COVID-19 case caused by sequence-confirmed SARS-CoV-2 B.1.1.529 (Omicron) variant in a Minnesota resident (patient A), the first such case identified in the state and one of the earliest identified in the United States. Patient A had attended a large indoor convention in New York, New York with approximately 53,000 attendees from 52 U.S jurisdictions and 30 foreign countries during November 19–21, 2021, and had close contact^†^ during 5 days with 29 fellow attendees. The convention required attendees to have received ≥1 COVID-19 vaccine dose and enforced mask-use while indoors. On November 22, these close contact attendees were directly and immediately notified by patient A of their exposure to SARS-CoV-2, and they sought testing over the next few days while quarantined or isolated. As part of the larger investigation into SARS-CoV-2 transmission at the convention, a subinvestigation was conducted during December by CDC, MDH, and respective state and local health departments to characterize the epidemiology of Omicron variant infection among this group of close contacts and determine the extent of secondary household transmission. Among 30 convention attendees that included patient A (the index patient) and the 29 other close contacts, 23 were interviewed, among whom all were fully vaccinated, including 11 (48%) who had received a booster dose; all 23 sought testing, and 16 (70%) received a positive SARS-CoV-2 test result. Fewer attendees who had received a booster dose before the convention received a positive test result (six of 11) compared with those who had not received a booster dose (10 of 12). The 16 attendees with positive test results had a total of 20 household contacts, 18 of whom sought testing after exposure; six received a positive test result for SARS-CoV-2. None of the persons with positive test results was hospitalized or died. There was limited convention-associated transmission identified outside of this cluster; the larger investigation included cases of both SARS-CoV-2 B.1.617.2 (Delta) and Omicron, and all Omicron cases were associated with this group (*1*). Data from this investigation reinforces the importance of COVID-19 booster doses in combination with early notification and other multicomponent prevention measures to limit transmission and prevent severe illness from Omicron and other SARS-CoV-2 variants.

Patient A flew to New York City on November 18 to attend a large convention with approximately 53,000 attendees. While in New York City, patient A stayed in a vacation rental with three other attendees and remained in close contact with 29 attendees during the 5-day visit. Patient A participated in several convention sessions and engaged in an informal schedule of social activities outside the convention that was shared with the 29 attendees, including mostly unmasked visits to restaurants, bars, clubs, and karaoke venues. The convention required proof of receipt of ≥1 COVID-19 vaccine dose, and mask use indoors at the convention was enforced for all attendees. Patient A was fully vaccinated in April 2021 and had received a booster dose on November 4. Patient A reported symptoms starting November 22, the day after the convention ended, upon returning home to Minnesota. The same day, patient A received a message from another attendee in this group who had received a positive at-home antigen test result that day (the first positive test result, but not the first reported symptom onset in the group). Patient A immediately informed all 29 attendees of their exposure so that they could take precautions and seek testing. Patient A sought real-time reverse transcription–polymerase chain reaction (RT-PCR) testing on November 23, which detected SARS-CoV-2 infection. A CDC-led investigation was initiated on December 3, 2021, in collaboration with MDH and the state and local health departments of the 29 other attendees.

## Investigation and Findings

Through a case investigation interview with patient A on November 30, contact tracing,^§^ and collaboration with various state and local health departments, the 29 attendees who had close contact with patient A during the event were identified. A questionnaire was developed to collect demographic, epidemiologic, and clinical information for the index patient and the 29 close contact attendees. In addition, a second questionnaire was developed to collect similar information from household contacts of any of these attendees (including patient A) with confirmed SARS-CoV-2 infection. During November 30–December 20, 2021, investigators conducted interviews with patient A, close contact attendees, and household contacts who did not attend the convention. This activity was reviewed by CDC and was conducted consistent with applicable federal law and CDC policy.^¶^

Among the 30 attendees (patient A and their 29 close contacts), 23 (77%) residing in 13 states** were interviewed; 12 (52%) were men, and the median age was 24 years. All 23 attendees had received a primary COVID-19 vaccination series (135–281 days before convention),^††^ with 11 (48%) having received a booster dose (six and five received the booster ≥14 days and <14 days, respectively, before the convention); one person reported a history of COVID-19 (Table 1).

**TABLE 1:** Characteristics of members of SARS-CoV-2 Omicron cluster who attended a New York City convention (N = 23) — 13 states,* November 18–December 20, 2021

Characteristic	No. (column %)	No. of attendees (row %)	
	Total (N = 23)^†^	SARS-CoV-2 test-positive^§^ (n = 16)	SARS-CoV-2 test-negative^¶^ (n = 7)
**Median age, yrs (range)**	**24 (21–41)**	**24 (22–41)**	**23 (21–31)**
**Male sex**	**12 (52)**	7 (58)	5 (42)
**No. days at the convention**			
3	**21 (91)**	14 (67)	7 (33)
2	**2 (9)**	2 (100)	0 (—)
**Reported mask use at the convention**			
Always	**20 (87)**	15 (75)	5 (25)
Sometimes	**3 (13)**	1 (33)	2 (67)
**History of previous COVID-19 infection**			
Yes	**1 (4)**	1 (100)	0 (—)
No	**22 (96)**	15 (68)	7 (32)
Primary vaccination series product**			
Pfizer-BioNTech	**14 (61)**	10 (71)	4 (29)
Moderna	**6 (26)**	3 (50)	3 (50)
Janssen (Johnson & Johnson)	**3 (13)**	3 (100)	0 (—)
**Received booster vaccine^††^**			
Yes	**11 (48)**	6 (55)	5 (45)
No	**12 (52)**	10 (83)	2 (17)
**Viral testing, self-tests**			
≥1 self-test (antigen)	**18 (78)**	13 (72)	5 (28)
≥1 positive result (n = 18)	**12 (67)**	12 (100)	0 (—)
**Viral testing, laboratory-based tests**			
≥1 laboratory-based test	**20 (87)**	14 (70)	6 (30)
≥1 positive result (n = 20)	**13 (65)**	13 (100)	0 (—)
**Location of test (n = 20)**			
Clinic	**14 (70)**	10 (71)	4 (29)
Pharmacy	**4 (20)**	2 (50)	2 (50)
Community testing center	**2 (10)**	2 (100)	0 (—)
**Type of test (n = 20)**			
Antigen test	**2 (10)**	1 (50)	1 (50)
RT-PCR (or other NAAT)	**18 (90)**	13 (72)	5 (28)
**Social activities outside of the convention**			
Outdoor sightseeing	**5 (22)**	3 (60)	2 (40)
Indoor sightseeing	**1 (4)**	1 (100)	0 (—)
Indoor restaurants	**19 (83)**	13 (68)	6 (32)
Outdoor restaurants	**7 (30)**	5 (71)	2 (29)
Bars	**18 (78)**	12 (67)	6 (33)
Nightclubs	**9 (39)**	6 (67)	3 (33)
Karaoke	**22 (96)**	16 (73)	6 (27)

**Abbreviations**: NAAT = nucleic acid amplification test; RT-PCR = reverse transcription–polymerase chain reaction.

* Arizona, California, Connecticut, Maryland, Massachusetts, Michigan, Minnesota, Missouri, New Jersey, New York, North Carolina, Texas, and Virginia.

^†^ Twenty-three of 30 attendees in this cluster were interviewed.

^§^ Positive result by antigen or NAAT/RT-PCR test.

^¶^ Negative result based on at least one viral test after exposure.

** https://www.cdc.gov/coronavirus/2019-ncov/vaccines/stay-up-to-date.html

^††^ All booster doses were administered as an mRNA COVID-19 vaccine.

Upon notification, all 23 interviewed attendees sought testing (15 within 2 days among 19 with known first test date), and 18 (78%) used one or more at-home antigen tests. Positive SARS-CoV-2 test results were received by 16 persons in 10 states^§§^ (attack rate = 70%), including two of three who used at-home antigen tests only, three of five who used laboratory-based tests only, and 11 of 15 who used both at-home antigen and laboratory-based tests. Five attendees’ specimens (from five states) were sequenced and were identified as Omicron variant sublineage BA.1 with no discernable difference. In addition, three household members of a single attendee had this same Omicron sequence confirmed, but the convention attendee in this household used an at-home antigen test only, and no sequence confirmation was available. Among 11 (48%) of the 23 interviewed attendees who had received a COVID-19 booster dose before the convention, six received a positive test result. Among 12 (52%) attendees who had not received a booster, 10 received a positive test result. All infected attendees reported at least one symptom (median = five) with symptom onset during November 21–26; seven reported symptom onset within 2 days of the final day of the convention. The median duration of symptoms was 11 days; the most commonly reported symptoms included nasal congestion, fatigue, cough, and sore throat. The median incubation period was 2–5 days (based on earliest and latest exposure). No hospitalizations or deaths were reported.

Overall, 21 (91%) of 23 interviewed attendees participated in the convention all 3 days. Twenty (87%) attendees reported mask-use “always” and three (13%) reported mask-use “sometimes” while at the convention (mostly removing mask to eat and take pictures). Attendees engaged in activities outside the convention, including karaoke, eating indoors, and visiting bars/clubs (Table 1). Among 14 interviewed attendees who stayed at accommodation A, nine received a positive test result, as did seven of nine who stayed in other accommodations (Figure). No attendee reported international travel before the convention.

**FIGURE:**
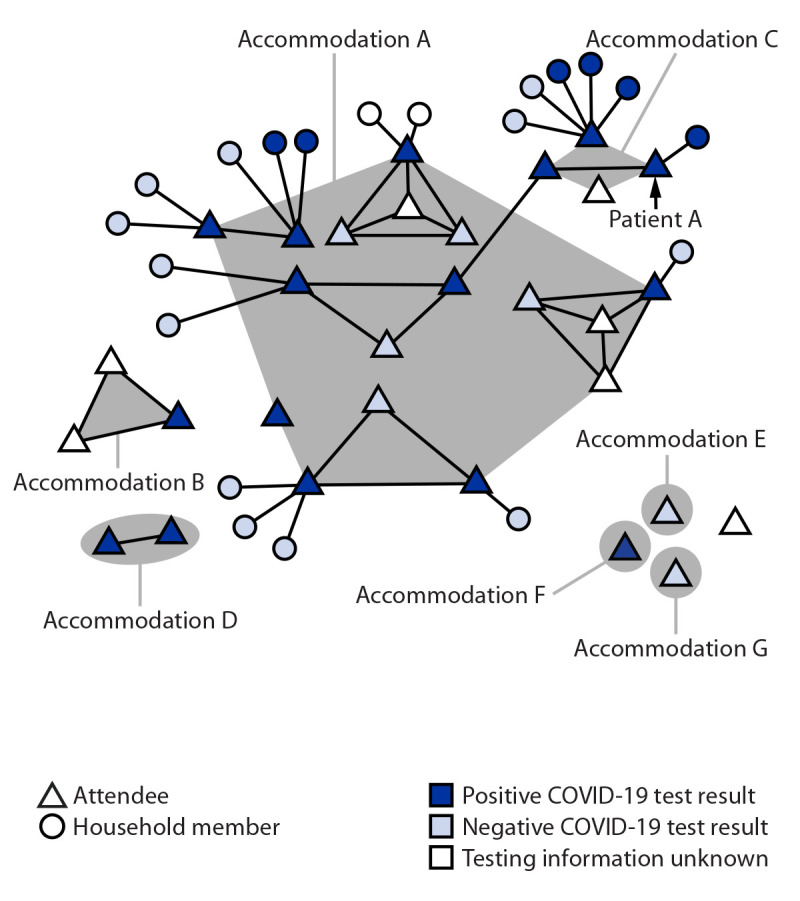
SARS-CoV-2 infections among a cluster of attendees of a New York City convention, by accommodation while in New York City and forward household transmission — 13 states,* November 18–December 20, 2021^†^ * Arizona, California, Connecticut, Maryland, Massachusetts, Michigan, Minnesota, Missouri, New Jersey, New York, North Carolina, Texas, and Virginia. ^†^ This figure was produced using MicrobeTrace. https://pubmed.ncbi.nlm.nih.gov/34492010/

Upon return home, the 16 attendees with positive test results exposed 20 household contacts (median age = 55 years) during their infectious period. Among household contacts, 11 (55%) were male, and none reported a previous history of COVID-19 (Table 2). Nineteen (95%) household contacts were fully vaccinated, and 10 (50%) had received a booster dose 2–94 days before exposure to the attendee (median = 27 days). Among 18 household contacts tested following exposure to an attendee with a positive SARS-CoV-2 test result, six (33%) received a positive result, including four who had received a booster dose >14 days before exposure. Symptom onsets ranged from November 26 to December 7 (median symptom duration = 13 days). Four patients reported fewer than five symptoms; the most commonly reported symptoms were nasal congestion, fatigue, cough, runny nose, and change in taste. The median incubation period was 1–5 days (based on earliest and latest exposure). No hospitalizations or deaths were reported.

**TABLE 2:** Characteristics of household contacts exposed to SARS-CoV-2 Omicron by a household member who acquired infection during trip to a New York City convention (N = 20)* — 10 states,^†^ November 18–December 20, 2021

Characteristic	No. (column %)	No. of household contacts (row %)	
	Total (N = 20)	SARS-CoV-2 test-positive^§^ (n = 6)	SARS-CoV-2 test-negative (n = 12)
**Median age, yrs (range)**	**55 (10–84)**	58 (20–84)	55 (10–63)
**Male sex**	**11 (55)**	4 (40)	6 (60)
**Relationship to convention attendee**			
Spouse or child	**2 (10)**	0 (—)	2 (100)
Parent	**11 (55)**	2 (22)	7 (78)
Grandparent	**2 (10)**	2 (100)	0 (—)
Sibling	**5 (25)**	2 (40)	3 (60)
**Primary vaccine series^¶^**			
Pfizer-BioNTech	**14 (70)**	4 (33)	8 (67)
Moderna	**3 (15)**	1 (33)	2 (67)
Janssen (Johnson & Johnson)	**2 (10)**	1 (50)	1 (50)
No vaccine	**1 (5)**	0 (—)	1 (100)
Received booster vaccine before exposure (n = 19)**			
Yes	**10 (53)**	4 (40)	6 (60)
No	**9 (47)**	2 (29)	5 (71)
**Viral testing**			
Rapid	**7 (35)**	4 (57)	3 (43)
Not rapid	**11 (55)**	2 (18)	9 (82)
Not applicable	**2 (10)**	0 (—)	0 (—)
**Exposures to convention attendee**			
Spent >15 minutes within 6 ft	**18 (90)**	6 (38)	10 (62)
Had face-to-face contact (within approximately 2 ft)	**14 (70)**	5 (38)	8 (62)
Spent any time within 6 ft while attendee was coughing or sneezing	**1 (5)**	1 (100)	0 (—)
Had direct contact with attendee (e.g., hug or kiss)	**11 (55)**	2 (22)	7 (78)
Slept in same bedroom	**1 (5)**	0 (—)	0 (—)
Shared a bathroom	**5 (25)**	2 (40)	3 (60)
Prepared food or share meal	**15 (75)**	5 (38)	8 (62)
Traveled in same vehicle (e.g., car, bus, or airplane), sitting within 6 ft	**10 (50)**	2 (22)	7 (78)
**No. of days exposed to convention attendee**			
1–2	**5 (25)**	2 (50)	2 (50)
3–5	**2 (10)**	0 (—)	2 (100)
≥6	**13 (65)**	4 (33)	8 (67)

**Abbreviations**: NAAT = nucleic acid amplification test; RT-PCR = reverse transcription–polymerase chain reaction.

* Two household members never sought testing.

^†^ Arizona, California, Connecticut, Massachusetts, Minnesota, Missouri, New Jersey, North Carolina, Texas, and Virginia.

^§^ Positive result by antigen or NAAT/RT-PCR test.

^¶^ https://www.cdc.gov/coronavirus/2019-ncov/vaccines/stay-up-to-date.html

** All booster doses were administered as an mRNA COVID-19 vaccine.

## Public Health Response

On December 2, 2021, MDH notified respective state and local health departments of four convention attendees with close contact to the index patient regarding potential exposure to the SARS-CoV-2 Omicron variant. That same day, CDC issued an Epidemic Information Exchange (Epi-X) to U.S. health departments to identify COVID-19 cases among event attendees. On December 6, CDC requested permission to conduct interviews with the index patient, close contact attendees, and their households; additional state and local health departments were notified as other close contacts were identified.

## Discussion

In this group of convention attendees with several days of close contact, SARS-CoV-2 Omicron variant attack rates were high, both among the attendees (70%) and among household contacts of infected attendees (33%). Among 16 cases that occurred within this attendee cluster, all persons had been vaccinated, as were the six household contacts who became infected. The high attack rates among fully vaccinated persons illustrate Omicron variant’s partial escape from vaccine-induced immunity (*2*); however, illness was relatively mild among this cohort, consistent with evidence that vaccinated persons with infections are less likely to experience serious illness. Potential contributing factors to the high attack rates include unmasked and prolonged contact in social settings and residential accommodations. This finding among this group contrasts with the observed overall transmission at the convention, which was relatively low; all Omicron cases identified from the larger investigation were associated with this group of close contact attendees. The low overall transmission at the convention is likely because of short interactions among participants in less confined space combined with high vaccination coverage, high prevalence of mask use, and high-efficiency particulate air filtration (*1*).

Among this attendee cluster with close unmasked contact and known booster status, a lower proportion of those who had received a COVID-19 booster dose received a positive test result (six of 11) compared with those who had not received a booster dose (10 of 12). Although this attendee cluster was small, the finding that prevalence was lower among those who had received a booster is consistent with other study findings (*2*–*4*). Vaccination, including booster doses,^¶¶^ reduces risk for infection; persons who have received a booster dose can still become infected, but they are less likely to experience severe illness, hospitalization, or death.

The findings in this report are subject to at least four limitations. First, testing for statistical significance was not performed because of the small sample size. Second, high use of at-home testing and time between positive test results and this investigation limited the availability of residual samples for sequencing; infections without sequencing data might have been caused by other variants. Third, seven close contact attendees never responded to requests for interview, which could have biased results. Finally, because at-home tests might have lower sensitivity than RT-PCR tests,*** some infections might have been undetected.

Early notification and testing with at-home antigen tests resulted in immediate quarantine or isolation and early diagnoses for the persons in this cluster, which might have reduced secondary attack rates in these households (*5*). Patient A notified the group after learning that another attendee was infected, 1–4 days after possible exposure, prompting contacts to seek testing, monitor for symptoms, and isolate or quarantine. The high use of at-home antigen tests suggests that persons with access to these tests will use them and share results. Although at-home antigen tests might have lower sensitivity than RT-PCR tests, broad access might result in earlier detection and notification, thus assisting in interrupting transmission. This investigation reinforces the importance of COVID-19 booster doses and early notification in combination with other multicomponent prevention measures to limit transmission and prevent severe illness from Omicron and other SARS-CoV-2 variants.

**Summary**

**What is already known about this topic?**

The SARS-CoV-2 Omicron variant is highly transmissible; is believed to have partial escape from infection- and vaccine-induced immunity; and is responsible for the recent rapid increase in U.S. cases.

**What is added by this report?**

Attack rates among a cohort of persons attending a convention were high, but lower among infected attendees’ household members. There were fewer infections among vaccinated attendees who had received a COVID-19 vaccine booster dose.

**What are the implications for public health practice?**

Data from this investigation reinforce the importance of COVID-19 booster doses and early notification in combination with other multicomponent prevention measures to limit transmission and prevent severe illness from Omicron and other SARS-CoV-2 variants.

## Footnotes

* These authors contributed equally to this report.

† Close contact is defined as being <6 feet away from an infected person (laboratory-confirmed or a clinical diagnosis) for a cumulative total of ≥15 minutes over a 24-hour period. https://www.cdc.gov/coronavirus/2019-ncov/php/contact-tracing/contact-tracing-plan/appendix.html#contact

§
https://www.cdc.gov/coronavirus/2019-ncov/php/contact-tracing/contact-tracing-plan/contact-tracing.html

¶ 45 C.F.R. part 46.102(l)(2); 21 C.F.R. part 56; 42 U.S.C. Sect. 241(d); 5 U.S.C. Sect. 552a; 44 U.S.C. Sect. 3501 et seq.

** Arizona, California, Connecticut, Maryland, Massachusetts, Michigan, Minnesota, Missouri, New Jersey, New York, North Carolina, Texas, and Virginia.

††
https://www.cdc.gov/coronavirus/2019-ncov/vaccines/stay-up-to-date.html

§§ Arizona, California, Connecticut, Massachusetts, Minnesota, Missouri, New Jersey, North Carolina, Texas, and Virginia.

¶¶
https://www.cdc.gov/coronavirus/2019-ncov/vaccines/booster-shot.html

***
https://www.cdc.gov/coronavirus/2019-ncov/testing/self-testing.html
